# Temperature Thresholds and Thermal Requirements for the Development of the Rice Leaf Folder, *Cnaphalocrocis medinalis*

**DOI:** 10.1673/031.013.9601

**Published:** 2013-09-30

**Authors:** Chintalapati Padmavathi, Gururaj Katti, V. Sailaja, A.P. Padmakumari, V. Jhansilakshmi, M. Prabhakar, Y.G. Prasad

**Affiliations:** 1 Directorate of Rice Research, Rajendranagar, Hyderabad, 500 030, India; 2 Central Research Institute for Dryland Agriculture, Santoshnagar, Hyderabad, 500 059, India

**Keywords:** biology, developmental thresholds, degree days, life table, pest forecast models

## Abstract

The rice leaf folder, *Cnaphalocrocis medinalis* Guenée (Lepidoptera: Pyralidae) is a predominant foliage feeder in all the rice ecosystems. The objective of this study was to examine the development of leaf folder at 7 constant temperatures (18, 20, 25, 30, 32, 34, 35° C) and to estimate temperature thresholds and thermal constants for the forecasting models based on heat accumulation units, which could be developed for use in forecasting. The developmental periods of different stages of rice leaf folder were reduced with increases in temperature from 18 to 34° C. The lower threshold temperatures of 11.0, 10.4, 12.8, and 11.1° C, and thermal constants of 69, 270, 106, and 455 degree days, were estimated by linear regression analysis for egg, larva, pupa, and total development, respectively. Based on the thermodynamic non-linear optimSSI model, intrinsic optimum temperatures for the development of egg, larva, and pupa were estimated at 28.9, 25.1 and 23.7° C, respectively. The upper and lower threshold temperatures were estimated as 36.4° C and 11.2° C for total development, indicating that the enzyme was half active and half inactive at these temperatures. These estimated thermal thresholds and degree days could be used to predict the leaf folder activity in the field for their effective management.

## Introduction

The rice leaf folder, *Cnaphalocrocis medinalis* Guenée (Lepidoptera: Pyralidae), is the most widely distributed and commonly found foliage feeder in all the rice growing tracts of Southeast Asia. An increase in *C. medinalis* population could be attributed to the large scale cultivation of high yielding varieties, application of fertilizers, and continuous use of insecticides leading to outbreak of this pest in several countries, including India (Khan et al. 1988; Shanmugam et al. 2006; Kaushik 2010). *C. medinalis* damages the rice plant throughout the crop growth period. The larvae fold the leaves longitudinally by stitching the leaf margins and feed by scraping the green mesophyll tissue from within the folded leaves. This feeding causes linear, pale white stripes that result in membranous patches (Fraenkel et al. 1981). Among the climatic factors, temperature is the most important, as it has profound influence on the development and survival of insects. The rate of insect development is affected by the temperature to which insects are exposed (Campbell et al. 1974). Insects require a certain amount of heat units (degree days) to develop from one life stage to the other (Gordan 1999). Quantification of the relationship between insect development and temperature is useful to predict the seasonal occurrence and population dynamics of the insects. The ability of an insect to develop at different temperatures is an important adaptation to survive under various climatic conditions (tropical, subtropical, and temperate). So far, there is no published report from India on the effect of constant temperatures on *C. medinalis*. Hence, in the present study, developmental periods of different stages of *C. medinalis* were examined at 7 constant temperatures to estimate the temperature thresholds and thermal requirements, which would be useful in developing models for predicting its distribution and abundance.

## Materials and Methods

### Study site

The study was conducted at the Directorate of Rice Research, Hyderabad, India. The climate in this region is predominantly semi-arid, with mean temperatures in the range of 22–42° C, and an average annual rainfall of 896 mm. Laboratory experiments were conducted using environmental chambers (MLR 350H, SANYO Electric Company,http://panasonic.net/sanyo/) set at constant relative humidity (60 ± 5%) and photoperiod (14:10 L:D).

### Stock culture of test insect

A stock culture of *C. medinalis* was maintained in the glasshouse at the Directorate of Rice Research on rice cultivar Taichung Native 1 (TN 1). Adults (10 to 15 pairs) collected from the field were kept for oviposition on 25-to 30-day-old TN 1 plants covered with a cylindrical mylar cage (45 cm height, 14 cm diameter) and were provided with honey (20% solution) as food. Every alternate day, pots were changed and the plants with eggs were shifted to wooden cages for hatching and further development. Larvae were shifted to fresh TN 1 plants when the green leaves got exhausted due to the folding and feeding. After completion of larval development, the pupae were transferred to a separate cage for adult emergence. Freshly emerged adults were collected daily, paired, and kept for oviposition. Egg laying was observed after 3 days of preoviposition period. These eggs were used in the experiments.

### Development and survival at different constant temperatures

Response to temperature was assessed by exposing *C. medinalis* eggs to 7 constant temperatures (18, 20, 25, 30, 32, 34, and 35° C) in separate experiments, 1 temperature at a time, and allowing the eggs to develop into adults. *C. medinalis* eggs (0–24 hr old) taken from the stock culture were placed in Petri dishes (9 cm diameter) at the rate of 10 per Petri dish in order to determine the development time. Fifty eggs at a time from the same cohort were kept on filter paper moistened to saturation with distilled water and sealed with Parafilm. The experiment was repeated thrice, each time with eggs from different cohorts. Thus, a total of 150 eggs were observed at each temperature. The Petri dishes with eggs were placed in environmental chambers maintained at different constant temperatures. The eggs were observed daily in order to determine the hatching rates and development time at each constant temperature. After hatching, each first instar larva was shifted to a separate Petri dish with 4 tender leaves of TN 1. Fresh leaves from 25- to 30-day-old TN 1 plants were provided as food daily until pupation. Petri dishes were checked every day until the larvae pupated. Moulting periods and dead larvae were recorded daily in order to determine the developmental periods and survival rates at each stage. Survival rates were calculated based on the number at the beginning and end of each stage. After pupation, pupae were transferred to individual glass tubes to observe adult emergence. The number of males and females emerged was counted to know the sex ratio. The emerged moths at each constant temperature were paired and released for oviposition on 25- to 30-day-old TN 1 plants covered with cylindrical mylar cage. The number of F1 eggs laid by each female was recorded to know the average fecundity. Since the hatching percentage was very low at 35° C, eggs were kept at ambient temperature (25 ± 5° C), and neonate larvae were shifted to the environmental chamber for further development after hatching.

### Temperature thresholds and thermal requirements

The degree day model (thermal summation model) was used to estimate the linear relationship between temperature and the rate of development of *C. medinalis* (Campbell et al. 1974). The reciprocal of developmental period for each stage was calculated to obtain the rate of development (1/day) at each temperature. Linear regressions were used to determine the relationship between developmental rate and temperature and to estimate intercept (a) and slope (b). These linear regression lines were extended to obtain the lower threshold for development (T_0_), which corresponds to the intersection with the abscissa (Davidson 1944; Wigglesworth 1972). After determining the lower temperature threshold for each stage, the thermal constant (the number of degree days required for complete development) was estimated from the reciprocals of the fitted regression line (b^-1^). Thus, the degree days required for the development of each life stage starting from egg to adult were estimated.

Sharpe-Schoolfield-Ikemoto (SSI) model, a non-linear thermodynamic model improved by Ikemoto (2005, 2008) and Shi et al. (2011) on the basis of the SS model developed by Sharpe and DeMichele (1977) and Schoolfield et al. (1981), was used. The SSI model expression is as follows:


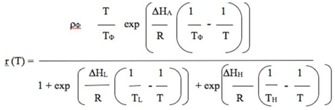


Where,

r = Mean development rate (1/day)

T = Absolute temperature (K) (237.15° K = 0° C)

R = Gas constant (1.987 cal/deg/mol)

ΔH_A_ = Enthalpy of activation of the reaction that is catalyzed by the enzyme (cal/mol)

ΔH_L_ = Change in enthalpy associated with low-temperature inactivation of the enzyme (cal/mol)

ΔH_H_ = Change in enthalpy associated with high-temperature inactivation of the enzyme (cal/mol)

T_L_ = Temperature at which the enzyme is ½ active and ½ low-temperature inactive (K)

T_H_ = Temperature at which the enzyme is ½ active and ½ high-temperature inactive (K)

T_Φ_ = Intrinsic optimum temperature at which the probability of enzyme being in the active state is maximal (K)

ρ_Φ_ = Development rate at the intrinsic optimum temperature TΦ (1/day) assuming no enzyme inactivation (days ^-1^)

In this model, the intrinsic optimum temperature (T_Φ_) for development is the most important thermal parameter. Ikemoto (2005) devised a program for estimating the parameters in the SSI model. Shi et al. (2011) modified this program and developed SSI-P, which runs on R statistical software (www.rproject.org) for faster estimation of the parameters. Ikemoto et al. (2012) further improved this program, creating OptimSSI-P by incorporating the optimization algorithm of Nelder and Mead (1965), wherein TΦ was estimated along with its confidence intervals. In this paper, OptimSSI program (version 2.7), which runs on R statistical software, version 2.15.0 (provided by Dr Peijian Shi, Chinese Academy of Sciences) was used to estimate the thermo-dynamic model parameters.

### Rate isomorphy

Rate isomorphy implies that the proportions of an organisms developmental stage durations are unaffected by temperature (van Rijn et al. 1995). Since the rate isomorphy is a consequence of equality among the lower developmental thresholds, statistical methods (Jarosik et al. 2002, 2004; Honek et al. 2003; Shi et al. 2010) were used for comparing the lower development thresholds. In this method, ratios of time spent in each developmental stage (proportion) at different constant temperatures were calculated from the data on the duration of development in a particular stage divided by the total pre-imaginal development (egg + larva + pupa). Analysis of covariance (ANCOVA) was performed using the arcsin square root of proportion as a response variable and temperature as a covariate. A significant (*p* < 0.05) increase or decrease in the proportion was considered as violation of the assumption of rate isomorphy (Kuang et al. 2012).

### Life table

A life table was constructed according to Ju et al. (2011) using Morris-Watt model (Moris 1963), which is explained in the following equation:


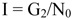


derived from survival rates SE, SL_1_, SL_2_, SL_3_, SL_4_, SL_5_, Sp, SA, FP_F_, P_♀_, where I is the population trend index; N1 is the number in next generation; No is the number in the current generation; S_E_, S_L1_, S_L2_, S_L3_, S_L4_, S_L5_, and S_P_ are the survival rates of eggs, 1^st^ instar, 2^nd^ instar, 3^rd^ instar, 4^th^ instar, 5^th^ instar, and pupae, respectively; SA is the survival rate of adults; F is the number of initial eggs; PF is the numberof average eggs laid by females; and P♀ is the female proportion of adults.

### Data analysis

The relationship between developmental period (by stage and instar) and temperature was analyzed following one-way ANOVA, and means were compared by Fisher's least significant difference procedures using SAS, version 9.2 (SAS 2008). Linear regressions were performed using SAS program.

## Results

### Development and survival

**Figure 1.**
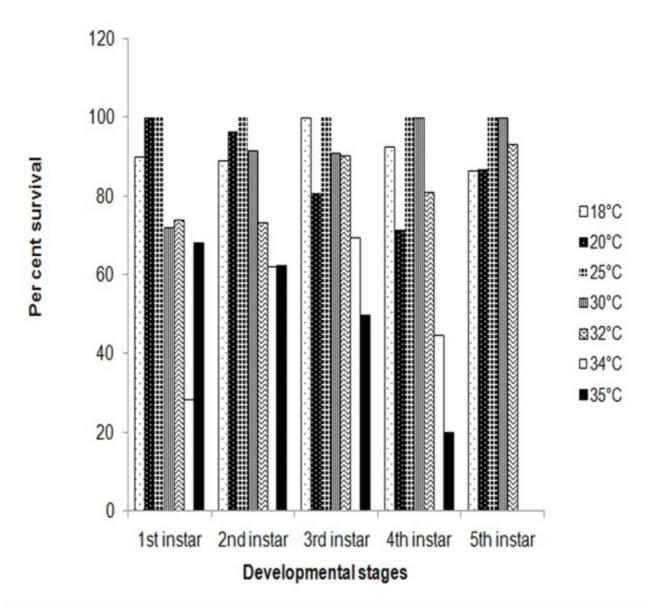
Percent survival of different larval instars of *Cnaphalocrocis medinalis* at 7 constant temperatures. High quality figures are available online.

**Figure 2.**
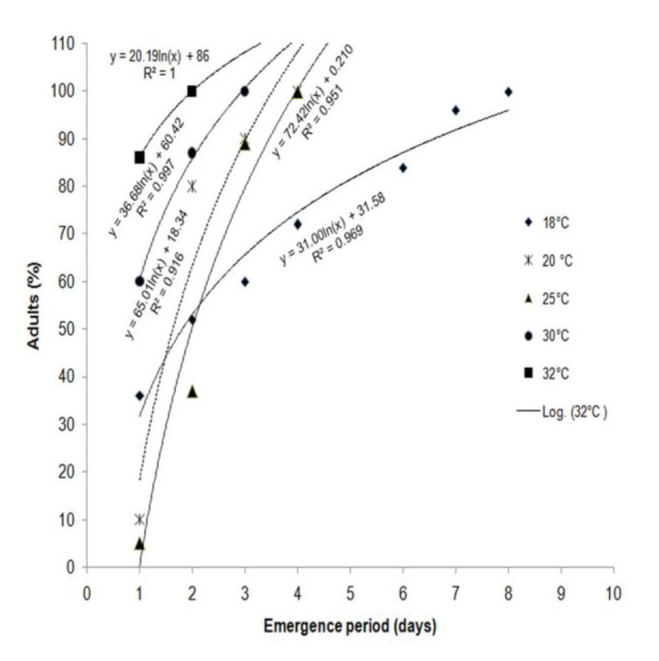
Cumulative emergence of *Cnaphalocrocis medinalis* adults at each constant temperature. High quality figures are available online.

Egg hatching varied from 16–82% in different constant temperatures, with minimum hatching at 35° C and maximum at 25° C. Although there was egg development at 35° C, neonate larvae died during eclosion due to the exposure to high temperature. The mean developmental time of eggs decreased from 9.51 days at 18° C to 3.10 days at 34° C, but increased to 3.58 days at 35° C, indicating a non-linear response at extreme temperatures (Table 1). First, second, and third instars also showed similar trends of development with increases in temperature. *C. medinalis* completed development from egg to adult at all 5 temperatures ranging from 18–32°C. Beyond 34° C, development took place only up to fourth instar, and the larvae could not survive thereafter. Survival of different larval instars of *C. medinalis* at 7 constant temperatures revealed that the survival was highest at 25° C, followed by 30° C (Figure 1). In the case of first instar larvae, 68% survival was observed at 35° C because of non-exposure of neonate larvae to higher temperature, as these were shifted from ambient temperature to environmental chamber after hatching. Adult emergence occurred at temperatures from 18– 32° C (Figure 2). The proportion of females in the total adult population varied from 50–64.10% in different temperatures. Cumulative adult emergence at these temperatures was best described by logarithmic curves with decreasing increments over time. The continuous lines in Figure 2 depict the curves best fitting the data. Emergence rates increased with increases in temperature, so that 100% emergence was attained earliest at 32° C (2 days), followed by 30° C (3 days). More than 50% emergence was observed on the first day at 32 and 30° C, and on the second and third days at 25 and 18° C, respectively. At 18° C, adult emergence was prolonged for about a week.

**Figure 3.**
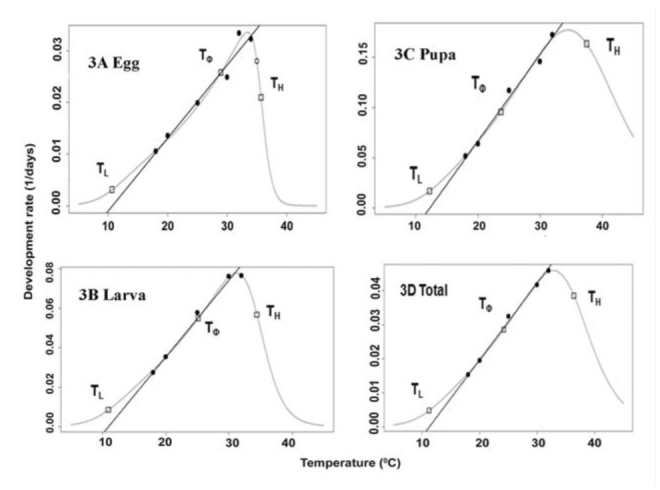
Linear and non-linear thermodynamic OptimSSI model fitted to the temperature dependent development of *Cnaphalocrocis medinalis*. Circles indicate data points. Open circles indicate data points outside the range of the linear model. The curved line indicates the values of developmental rate predicted by the OptimSSI model, whereas the straight line denotes the values of linear fitting. The 3 open squares denote the predicted mean developmental rates at T^L^, T^Φ^, and T^H^. High quality figures are available online.

### Temperature thresholds and thermal requirements

Extrapolation of linear regression lines of developmental rate and temperature showed that the lower threshold temperatures (T0) for each life stage of *C. medinalis* were between 5.0 and 13.0° C (Table 2). The T0 of 11.0° C, 10.4° C and 12.8° C were estimated for the development of eggs, larvae, and pupae, respectively. The T0 varied between larval instars, having the lowest value (5.0° C) for 3^rd^ instar and the highest (13.0° C) for 4^th^ instar (Table 2). The estimated thermal constants (K) for egg, larva and pupa were 69, 270, and 106 degree days, respectively. The T0 and thermal constant for total development (egg to adult) were 11.1° C and 455 degree days, respectively.

A thermodynamic non-linear model (Optim SSI) was used to estimate the intrinsic optimum temperature (T_Φ_) for development. Based on enzyme activity, other parameters were also estimated. In this program, the results of a linearized formula (Ikemoto and Takai 2000) based on the reduced major axis method were also obtained. Lower development threshold values of 10.7, 10.9, 12.2, and 11.2° C, with sum of effective temperatures of 71, 257, 116, and 445 degree days, were estimated for eggs, larvae, pupae, and total development, respectively (Table 3, Figure 3). The intrinsic optimum temperature for the development of eggs, larvae, pupae, and eggs to adult was estimated at 28.9, 25.1, 23.7, and 24.2° C, respectively. The lower and upper confidence limits for each parameter and each stage are presented in Table 4. Upper threshold temperatures (T_H_) for eggs, larvae, pupae, and total development were estimated at 35.7, 34.4, 37.5, and 36.4° C, respectively (Table 4).

### Rate isomorphy

Estimates of parameters and standard errors using the Ikemoto and Takai method (2000) to test the rate isomorphy of immature stages of *C. medinalis* are given in Table 2. ANCOVA results showed that the interaction was significant (*p* < 0.0001). Thus, the slopes of the regression lines of 3 immature stages, i.e., egg, larva, and pupa, were different, and hence rate isomorphy was not evident.

### Life table and population trend index

Based on the survival rate, sex ratio, and fecundity, a life table was constructed for *C. medinalis* in order to know the potential of population growth (Table 5). In the table, the standard number of eggs was taken as the initial count (150), and hatching rate of eggs(S_egg_), survival rate of the five instars of larvae (S_1_, S_2_, S_3_, S_4_, S_5_), and female proportion were based on the actual data from this study. Population trend index (I) was calculated for all the temperatures and was found to be highest at 30° C (14.46), followed by 25° C (12.16) and 32° C (10.6), indicating that temperatures of 25–30°C were most favorable for *C. medinalis* population growth.

## Discussion

Temperature is the most important and critical abiotic factor exerting profound influence on the development of insects. The relationship between temperature and rate of development is crucial, as it influences insect biology, distribution, and abundance (Braman et al. 1984; Legg et al. 2000; Tobin et al. 2003). Development of *C. medinalis* at 7 constant temperatures revealed decreases in developmental time from egg to adult with increases in temperature (Table 1). At 32° C, total development was completed in 21.8 days, while it took 65.4 days at 18° C. A similar decreasing trend was observed in different stages from 18–34° C, but the duration increased at 35° C. However, *C. medinalis* could not complete its development at 34 and 35° C, as larvae could not survive beyond 4^th^ instar. Earlier studies on *C. medinalis* from different regions reported variations in the developmental periods of eggs, larvae, and pupae, and the total developmental periods (egg to adult) were in the range of 24–41 days (Lingappa 1972; Yadava et al. 1972; Velusamy and Subramaniam 1974; Godase and Dumbre 1982; Padmavathi et al. 2006), but all these results were based on experiments carried out under ambient temperatures. A similar decrease in the developmental period with an increase in constant temperatures was reported in the case of other pyralid rice stem borers, e.g., *Chilo suppressalis*, *C. polychrysa*, *C partellus*, *Scirpophaga incertulas*, and *S innotata* (Rahman and Khalequzzaman 2004). In our study, the survival of *C. medinalis* larval instars was highest at 25° C, followed by 30° C (Figure 1). Sato and Kishino (1978) also reported the highest survival of *C. medinalis* at 25° C and observed an increase in mortality with a decrease or increase in temperature, indicating a non-linear relationship. Cumulative emergence rates at constant temperatures revealed that 100% of adults emerged on day 1 at 32° C, while the emergence was prolonged at 18° C, indicating faster development with increased temperature (Figure 2).

Estimation of lower threshold temperatures and thermal constants for different stages of *C. medinalis* from linear regressions revealed lower development threshold (T0) values at 11.0, 10.4, and 12.8° C for eggs, larvae, and pupae, respectively (Table 2). Thermal constants of 69,270 and 106 degree days were calculated for eggs, larvae, and pupae, respectively. Intrinsic optimum temperature (TΦ) of 24.2° C was obtained for the total development from egg to adult, suggesting the maximal active state enzymes involved in the developmental process. The upper (T_H_) and lower (TL) threshold temperatures were estimated at 36.4 and 11.2° C for total development, suggesting that the hypothetical enzyme was half active and half inactive at these thresholds. This could be one of the reasons for the incomplete development of the larval stage at 34 and 35° C. The intrinsic optimum temperature along with its confidence interval could be used as an indicator for the geographical distribution and place of origin of related species (Ikemoto 2003). This is also a potential tool for the construction of a phylogenetic tree within a taxon (Ikemoto et al. 2012).

The present results are in accordance with Graf et al. (1992) who developed a simulation model by establishing a common developmental threshold of 12.4° C and thermal constants of 70, 280, 110, and 140 degree days for eggs, larvae, pupae, and adults of *C. medinalis*, respectively. However, Wada (1979) reported that the frequency of molting in the larval stage of *C. medinalis* changes with the growing stage of the rice plant, and suggested that the thermal constant is influenced not only by temperature, but also by the host plant. In another field study, Sato and Kishino (1978) found that in the population of *C. medinalis* from Sapporo, Japan, the thresholds of development for eggs, larvae, pupae, and total development, were 11.2, 11.9, 13.3, and 12.0° C, respectively.

Analogous studies in another pyralid, *Chilo partellus*, revealed that it took 588.34 degree days above 17.6° C for the completion of all developmental stages (Jalali and Singh 2001). Thermal constants of 705.56, 725.32, 703.30, 556.59, 655.34, and 837.95 degree days were reported for *C. polychrysa*, *C. suppressalis*, *C. partellus*, *Scirpophaga incertulas*, *S. innotata*, and *Sesamia inferens*, respectively, with the mean developmental zero of 7.70–10.19° C in different species (Rahman and Khalequzzaman 2004). Jarosik et al. (2011) reported that closely related species shared similar thermal requirements because of common intrinsic optimum temperature and duration of development by comparing several phenology models. This intrinsic optimum temperature could be used as an indicator for classifying phylogenetic relatedness (Ikemoto 2005). In the natural fluctuating day and night environment, *C. medinalis* may be able to develop and survive at higher temperatures than observed in the present constant temperature studies. Threshold temperature and effective accumulated temperatures of 10.07° C and 558.36degree days were reported for the whole generation of green semi-looper, *Naranga aenescens*, and no obvious differences were observed in their threshold temperatures and effective accumulated temperature between constant temperature studies in the laboratory and alternating temperature studies in paddy fields (Lu et al. 2002). The estimated temperature thresholds and thermal constants are useful in the prediction of population peaks (Taveras et al. 2004), to identify optimal time of insecticide application (Tolley and Robinson 1986), to estimate intrinsic rate of natural population increase (Kinjo and Arakaki 2002), to develop a forecasting system to monitor the adult emergence and flight activity (Ahmad and Ali 1985), and to develop phenology models (Jarosik et al. 2011). The threshold temperatures estimated, particularly T_L_ and T_H_, are also useful to study the impact of climate change on the distribution of a species (Kocmankova et al. 2010).

Rate isomorphy testing by ANCOVA revealed a significant difference among the slopes of linear regressions of different developmental stages of *C. medinalis*, indicating that the lower development thresholds were different. Possible reasons for the violation of the general rule of developmental isomorphy could be the coarse estimates of development at high temperatures or mortality at low temperatures (Jarosik et al. 2002).

Life tables are powerful tools for understanding the changes in a population during different stages of growth, and are governed by a number of biotic and abiotic factors. The role of biotic factors in regulating the population of *C. medinalis* in the field was reported by Padmavathi et al. (2008) while constructing and analyzing mortality and fertility life tables. Data from our study indicated that temperatures between 25 and 30° C were favorablefor the survival and multiplication of leaf folder populations.

A comparative analysis of the published data sets indicated that the lower threshold value obtained in this study (11.1) was similar to the report by Chang and Wu (1988). However, higher T_0_ values were reported from Japan and the Northern part of China (Table 6). Thus, the variation in lower threshold and thermal constant among leaf folder populations from different geographical areas could be attributed to multiple factors, such as experimental conditions, host-plant quality, thermal adaptations to different geographical areas, and the method of estimation (Marchioro and Foerster 2011).

In the present study, 24.2° C was estimated as the intrinsic optimum temperature for the development of *C. medinalis*, with a thermal constant of 445 degree days. Lower (T_L_) and upper (T_H_) thresholds were estimated at 11.2° C and 36.4° C, respectively. The estimated temperature thresholds and thermal constants are potential indicators of the distribution and abundance of *C. medinalis*. These parameters are useful in developing an insect phenology model for predicting population dynamics for pest management under field conditions.

**Table 1.**
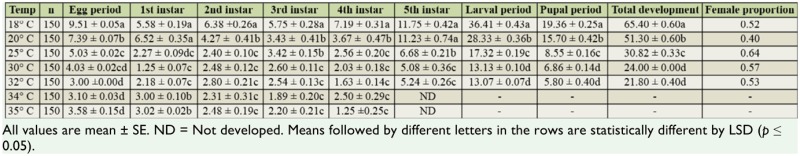
Developmental time (days) of different stages of rice leaf folder, *Cnaphalocrocis medinalis*, at 7 constant temperatures.

**Table 2.**
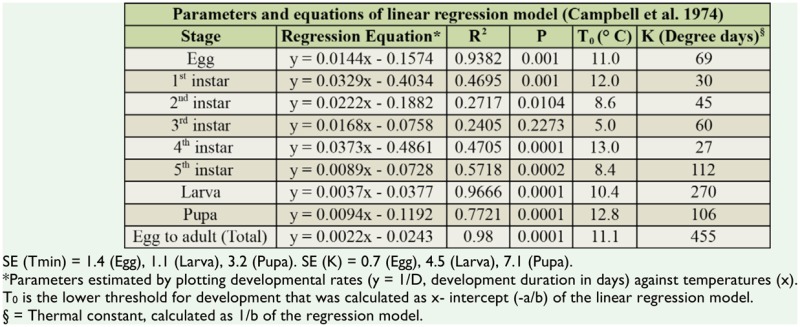
Linear regression equations, lower developmental thresholds, and thermal constants of each developmental stage of *Cnaphalocrocis medinalis*.

**Table 3.**
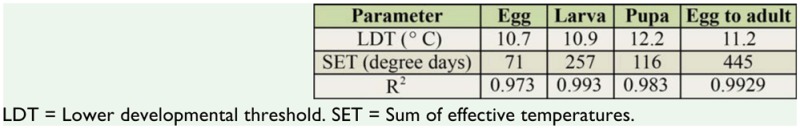
Estimations of lower developmental thresholds and sum of effective temperatures by Ikemoto and Takai's (2000) linear model.

**Table 4.**
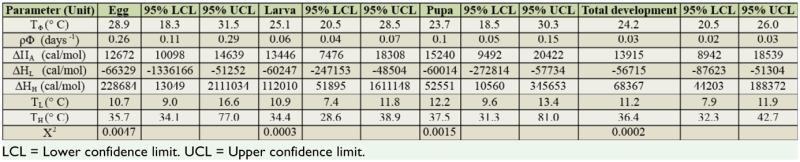
Parameters of non- linear thermodynamic model (OptimSSI) for different stages of *Cnaphalocrocis medinalis*.

**Table 5.**
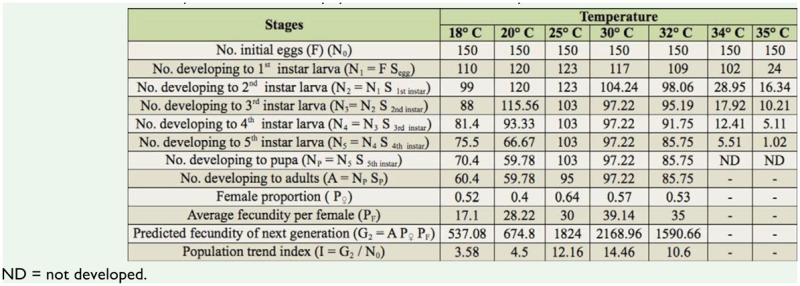
Life table of *Cnaphalocrocis medinalis* population at 7 constant temperatures.

**Table 6.**
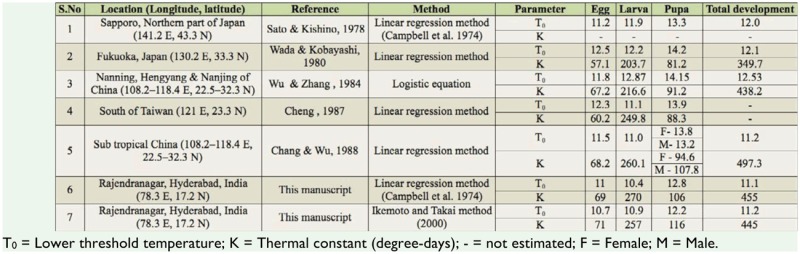
Comparative thermal requirements for life stages of *Cnaphalocrocis medinalis* in Southeast Asia.

## Abbreviations:

SSI,: Sharpe-Schoolfield-Ikemoto
TN,: Taichung Native
